# Can ultrasound novices develop image acquisition skills after reviewing online ultrasound modules?

**DOI:** 10.1186/s12909-021-02612-z

**Published:** 2021-03-20

**Authors:** Elaine Situ-LaCasse, Josie Acuña, Dang Huynh, Richard Amini, Steven Irving, Kara Samsel, Asad E. Patanwala, David E. Biffar, Srikar Adhikari

**Affiliations:** 1 University of Arizona, College of Medicine & Banner University Medical Center - Tucson, Department of Emergency Medicine, PO Box 245057, Tucson, AZ 85724 USA; 2 Tucson Medical Center, Department of Emergency Medicine, Tucson, AZ USA; 3 College of Medicine, Department of Emergency Medicine, University of Arizona, Tucson, AZ USA; 4 Medical Center Hospital, Odessa, TX USA; 5 Department of Emergency Medicine, Texas Tech University Health Sciences Center, El Paso, TX USA; 6 University of Sydney, Sydney School of Pharmacy, Sydney, Australia; 7 Arizona Simulation Technology and Education Center – University of Arizona Health Sciences, Tucson, AZ USA

**Keywords:** Point-of-care ultrasound, Medical student ultrasound education, Simulation, Ultrasound education, Asynchronous learning

## Abstract

**Background:**

Point-of-care ultrasound is becoming a ubiquitous diagnostic tool, and there has been increasing interest to teach novice practitioners. One of the challenges is the scarcity of qualified instructors, and with COVID-19, another challenge is the difficulty with social distancing between learners and educators. The purpose of our study was to determine if ultrasound-naïve operators can learn ultrasound techniques and develop the psychomotor skills to acquire ultrasound images after reviewing SonoSim® online modules.

**Methods:**

This was a prospective study evaluating first-year medical students. Medical students were asked to complete four SonoSim® online modules (aorta/IVC, cardiac, renal, and superficial). They were subsequently asked to perform ultrasound examinations on standardized patients utilizing the learned techniques/skills in the online modules. Emergency Ultrasound-trained physicians evaluated medical students’ sonographic skills in image acquisition quality, image acquisition difficulty, and overall performance. Data are presented as means and percentages with standard deviation. All *P* values are based on 2-tailed tests of significance.

**Results:**

Total of 44 medical students participated in the study. All (100%) students completed the hands-on skills evaluation with a median score of 83.7% (IQR 76.7–88.4%). Thirty-three medical students completed all the online modules and quizzes with median score of 87.5% (IQR 83.8–91.3%). There was a positive association between module quiz performance and the hands-on skills performance (R-squared = 0.45; *p* < 0.001). There was no statistically significant association between module performance and hands-on performance for any of the four categories individually. In all four categories, the evaluators’ observation of the medical students’ difficulty obtaining views correlated with hands-on performance scores.

**Conclusions:**

Our study findings suggest that ultrasound-naïve medical students can develop basic hands-on skills in image acquisition after reviewing online modules.

**Supplementary Information:**

The online version contains supplementary material available at 10.1186/s12909-021-02612-z.

## Background

Point-of-care ultrasound (POCUS) is rapidly becoming a more ubiquitous diagnostic and treatment tool in various medical specialties. Training in this imaging modality is required in emergency medicine residency education, but POCUS is extending into other specialties and settings. The increasing interest in this modality is driving the earlier integration of ultrasound education into the medical school curriculum. Medical students are learning how to use ultrasound to augment their physical examination skills, and they are seeing the utility in this imaging modality for their future careers [1].

In addition to didactics, one of the critical components of ultrasound education is teaching the psychomotor skills in performing ultrasound examinations. This development of ultrasound muscle memory typically requires hands-on practice in the form of in-person proctoring by an ultrasound expert. However, one of the challenges with educating an ever-growing base of learners is the scarcity of resources, especially qualified teachers and equipment. Methods for addressing this problem vary widely, from flipped classroom instruction to using telemedicine platforms [2]. Fuchs et al. showed that medical students can learn cardiac ultrasound through electronic learning platforms as well as their validated bedside in-person cardiac course [3]. These methods for teaching traditionally hands-on examinations/procedures are especially important in today’s COVID-19 (Coronavirus Disease 2019) pandemic. With social distancing and cancellation of in-person teaching sessions, direct observation and in-person teaching of novices have become difficult and perhaps, non-existent. Therefore, ultrasound educators must be creative in creating an alternative way to deliver both the medical knowledge-based and psychomotor learning.

Ultrasound education platforms are constantly evolving and improving their products to meet the growing demand. Several of these platforms are simulation-based, virtual reality-based, or web-based modules [4–6]. It is unclear if these tools can replace more traditional teaching methods of didactics and in-person hands-on training sessions [7, 8]. Because of this, we wanted to explore the effectiveness of an online training program to learn hands-on skills. This online training program is comprised of various modules for frequently used POCUS examinations. Each module is comprised of a lecture section demonstrating the technique of POCUS examinations and a multiple-choice exam at the end of each module to test knowledge retention. Students’ progression and test scores are viewable by the instructor.

To our knowledge, there are no studies exploring learning ultrasound and hands-on skills solely based on online modules, only those that explore purely online modules without a hands-on assessment or more traditional methods of a didactics component and hands-on training [8–10]. Therefore, our objective for this study was to determine if ultrasound-naïve operators (first-year medical students) can learn ultrasound technique and develop the psychomotor skills necessary to acquire ultrasound images after only reviewing online modules.

## Methods

### Study aim, design, and setting

The aim for the study for this study was to determine if ultrasound-naïve operators can learn ultrasound technique and develop the psychomotor skills for ultrasound image acquisition after reviewing online modules. This was a prospective cohort educational study conducted at an academic medical center. The study falls under our College of Medicine’s umbrella institutional review board (IRB) as approved education research according to the pre-determined requirements of the College of Medicine’s original IRB approval. Inclusion criteria were: first-year medical students who were ultrasound-naïve, which was ultimately, the study population. Participants gave informed consent. Participation in this study was voluntary, and it is not a part of their standard medical school curriculum. Sample size was solely determined by the number of students who volunteered to be in the study and not determined a priori. It was presumed that this sample of ultrasound-naïve students was representative of the first-year medical students as a whole, based on their ultrasound experience. Data collection and analysis were performed in September 2017.

### Study protocol

We assigned four core anatomy and physiology ultrasound examination online modules to ultrasound-naïve medical students (aorta/IVC, cardiac, renal, and superficial) and tested their hands-on skills after they completed modules which contained education on indications, normal sonoanatomy, ultrasound technique along with online quizzes. Students had three weeks to complete approximately 9 h of online learning and quizzes. The week after the completion deadline, the students completed in-person hands-on evaluations of image acquisition and perceived learner difficulty by Emergency Medicine Ultrasound-trained faculty and fellows. A short questionnaire was also administered at the end of the hands-on evaluation session. The previously piloted and validated paper questionnaire included items pertaining to opinions regarding the educational intervention and their image acquisition confidence level on a 1 to 10 scale [11, 12].

### Online ultrasound learning modules

The SonoSim® Ultrasound Training Solution was chosen for our study because it was already purchased by our simulation center for ultrasound education. Because there was only one physical system and one sham transducer, it was not possible to train several students at the same time using the sham transducer. So, we opted for an online-only training plan.

The online modules were from SonoSim® Ultrasound Training Solution. SonoSim® Ultrasound Training Solution (Santa Monica, CA) is an education and technology company that markets ultrasound training modules with integrated simulated hands-on ultrasound training, didactic instruction, and assessment. The integrated simulated hands-on ultrasound training (sham ultrasound probe for hands-on practice within the SonoSim application/laptop) was not used for this study. This ultrasound training system is designed to provide similar training as traditional in-person training sessions. Four ultrasound anatomy and physiology modules were pre-selected for our study, and volunteer medical students were given three weeks to review these four pre-selected online SonoSim® modules and to complete the associated quizzes. SonoSim® Ultrasound Training Solution modules take approximately 2 h each to complete. Each module uses still images, videos, and narration to teach learners basic anatomy and physiology of the particular body part along with sonographic anatomy and physiology. The modules also cover ultrasound scanning technique with narrated videos demonstrating probe placement and movement. There are in-module and end-of-module assessments. Module progress and end-of-module examination performance can be seen by the instructor. Links to more detailed module descriptions are included in the supplementary materials.

### Assessment

After completion of the training modules, in the following week, each medical student was asked to acquire certain ultrasound views on standardized patients (Additional file 1). Assessors for this course were department of Emergency Medicine Ultrasound fellowship-trained faculty and fellows with expertise in bedside ultrasound. The primary outcome measure was performance in image acquisition. Assessment was conducted using a set rubric akin to the Likert scale (Additional file 1). The students were asked to acquire sonographic images of the aorta/IVC, heart, kidneys, and soft tissue. The assessors could give up to 2 points depending on the requested image or question. The level of difficulty each student had with the four hands-on components was rated by the evaluator based on their perception of the learners’ ease at obtaining particular views. At the end of the hands-on assessment, each student completed a three-question survey regarding their experience. Additional Files 1 and 2 are the hands-on evaluation form and the medical student survey.

### Data analysis

All analyses were performed using Stata 11 (StataCorp LP, College Station, TX). Data are presented as means and percentages with standard deviation. All *P* values are based on 2-tailed t-tests of significance. *P* values of less than 0.05 are considered statistically significant.

## Results

A total of 44 first-year medical students enrolled in the study. There were 23 females (52.3%) and 21 males (47.7%). Ninety-five percent (42/44) of the students reviewed all of the SonoSim® training modules, while 75% (33/44) reviewed all the online modules and completed the associated quizzes receiving a median score of 87.5% (IQR 83.8–91.3%). All (100%) students participated in the hands-on skills evaluation with a median score of 83.7% (IQR 76.7–88.4%). There was a positive association between performance on the module quizzes and performance during the hands-on skills evaluation (R-squared = 0.45; *p* < 0.001) (Table 1). When analyzed individually, there was no statistically significant association between module performance and hands-on performance for any of the categories, except for aorta/IVC (Table 1). Table 2 shows that the students who did not complete all the modules consistently scored lower than those students who completed the modules in their entirety. On all four categories, the evaluators’ observation of the medical students’ difficulty obtaining views correlated with their hands-on performance scores (Table 3). Additional File 3 shows the collected data points in more detail and Additional File 4 shows more data analysis. Table 4 shows the results of the paper questionnaire that was completed by the students.

**Table 1:** SonoSim® Online Module Performance Scores and Hands-On Assessment Scores

	Average Online Module Score (%)	Average Hands-on Assessment Score of Students Who Completed 100% of the Particular Module (%)	***P***-value
**Overall Score**	87.5 ± 5.0 (*n* = 33)	82.2 ± 12.1 (*n* = 33)	0.000
**Aorta/IVC**	87.8 ± 7.5 (*n* = 39)	83.8 ± 13.8 (*n* = 39)	0.011
**Cardiac**	86.3 ± 7.3 (*n* = 34)	75.7 ± 20.2 (*n* = 34)	0.086
**Renal**	84.2 ± 6.2 (*n* = 37)	81.9 ± 14.6 (*n* = 37)	0.162
**Superficial**	88.8 ± 7.3 (*n* = 38)	91 ± 10.2 (*n* = 38)	0.193

**Table 2:** Hands-On Assessment Scores of Participants Who Completed 100% of Modules Versus Participants Who Did Not Complete All the Modules

	Average Hands-on Assessment Score of Students Who Completed 100% of the Particular Module (%)	Average Hands-on Assessment Score of Students Who Did NOT Complete 100% of the Particular Module (%)
**Overall Score**	82.2 ± 12.1 (*n* = 33)	74.6 ± 14.8 (*n* = 11)
**Aorta/IVC**	83.8 ± 13.8 (*n* = 39)	71.7 ± 22.5 (*n* = 5)
**Cardiac**	75.7 ± 20.2 (*n* = 34)	64.6 ± 18.9 (*n* = 10)
**Renal**	81.9 ± 14.6 (*n* = 37)	76.9 ± 21.3 (*n* = 7)
**Superficial**	91 ± 10.2 (n = 38)	78.6 ± 21.7 (*n* = 6)

**Table 3:** Average Hands-on Assessment Scores for All Students and Perceived Level of Difficulty by Evaluator

	Average Hands-on Assessment Score for ALL Students (%) (***n*** = 44)	Average Perceived Level of Difficulty During Hands-on Assessment [1 (low) to 5 (high)] (***n*** = 44)	***P***-value
**Overall Score**	82.4 ± 12.1	–	–
**Aorta**	79.6 ± 17.3	2.5 ± 1.0	0.002
**IVC**	84.1 ± 23.3	2.5 ± 0.9	0.000
**Cardiac**	73.1 ± 20.3	3 ± 1.0	0.002
**Renal**	81.1 ± 15.7	3 ± 1.0	0.002
**Superficial**	89.3 ± 12.8	1.9 ± 0.9	0.000

**Table 4:** Post-Assessment Survey Results

Survey Question	Percentage of students of students who responded:									
	Strongly Agree		Somewhat Agree		Neither Agree nor Disagree		Somewhat Disagree		Strong Disagree	
**“SonoSim**® **education modules are a good start to learning sonographic anatomy.”**	**84.1**		**15.9**		**0**		**0**		**0**	
**“SonoSim**® **education modules are adequate to learning ultrasound scanning technique.”**	**43.2**		**40.9**		**6.8**		**9.1**		**0**	
	**1** **(low confidence)**	**2**	**3**	**4**	**5**	**6**	**7**	**8**	**9**	**10** **(high confidence)**
**Rate level of confidence in acquiring ultrasound images.**	0	0	2.3	4.5	22.7	18.2	31.8	13.6	6.8	0

## Discussion

As point-of-care ultrasound continues to permeate through nearly every specialty, it is important for software development, simulation developers, and any ultrasound education source to be mindful of key components required to cultivate ultrasound skills. Previous literature suggests that core psychomotor skills and fundamental motor skills are developed and subsequently applied during each ultrasound examination performed [13]. These skills are used by the operator to move and manipulate the transducer in response to sensory information such as the image on the screen or the feel of a chest wall rib. However, the traditional model of ultrasound training is in-person, hands-on practice either with live models or costly simulators [1, 4, 7, 14]. These in-person training sessions are resource-intensive, requiring expensive equipment and highly trained instructors. Time, effort, financial, and space restraints are also challenges educators face [1, 15]. To mitigate some of these issues, we sought to find alternatives in point-of-care ultrasound education. Because of the COVID-19 pandemic, there is no better time to explore various platforms to maintain consistent education delivery and high-quality learning, while protecting the health of learners and educators. Ultrasound educators, in particular, are brainstorming creative ways to ensure their ultrasound novices are receiving both the medical knowledge and the psychomotor training they need to develop the muscle memory required for competency [14].

In our study, we chose to use the SonoSim® Ultrasound Training Solution, which was already purchased by our simulation center for ultrasound education. Since there was only one physical system and one sham transducer, it was not possible to train several students at the same time using the sham transducer. Instead, we opted to assign four core ultrasound examination online modules: aorta/IVC, cardiac, renal, and superficial, to ultrasound-naïve medical students and tested their hands-on skills after they completed modules which contained education on indications, normal sonoanatomy, ultrasound technique along with online quizzes. We chose to test the students at three weeks because clinical skills retention rates are established to show significant decline between weeks 6 and 12, and we wanted to avoid time as a factor in poor hands-on assessment outcomes [16]. This conclusion was also supported by Bosse et al’s study, which showed frequent, repetitive skills training sessions and feedback result in a strong improvement of early procedural skill acquisition [17]. Studies which include a robust hands-on component show more lasting results of skills retention on the scale of months to a year [18, 19]. However, because our study is only assessing knowledge retention from online didactics, we opted to assess the students shortly after they reviewed the online modules.

Based on our results, ultrasound-naïve medical students who performed well on the online ultrasound module quizzes overall also performed well on the hands-on assessments overall. However, when evaluated separately, only the aorta/IVC module performance correlated with the hands-on performance for that exam type. The evaluation of all 44 students’ hands-on assessment scores revealed that their review of the online modules translated into their ability to acquire various images on a standardized patient. Evaluators’ perception of the students’ difficulty acquiring images correlated to their hands-on scores.

The range of hands-on assessment scores is 46.5 to 97.7%. This range includes students who did not complete all the online modules, but for those who did complete all of the modules and their corresponding quizzes, the average hands-on score was 82.2%. Table 2 shows the differences in hands-on scores of both students who completed 100% of them modules and those who did not. Consistently, the students who did not complete the specific module performed worse than the students who did. Some students reviewed the content of the modules but did not complete the associated quizzes. It appears that those who completed all modules and quizzes performed better, showing a testing effect. This shows that those who learned sonographic anatomy and sonographic technique from the modules were able to acquire ultrasound images on live patients.

Literature has already shown that with online materials, in-person didactic teaching, and hands-on practice sessions, medical student ultrasound novices can achieve a level of competency in POCUS [20]. Our study suggests that perhaps medical students can learn a significant amount of ultrasound from asynchronous learning materials. Although our study did not evaluate the hands-on practice capabilities of the SonoSim® Ultrasound Training Solution, we believe that the online modules are a good start to learning ultrasound and a useful tool for repetition and knowledge retention. Because the modules include narrated videos demonstrating practical, hands-on placement of ultrasound transducers on live models, this is akin to in-person live demonstrations. These videos provide a visuo-spatial approach to bedside ultrasound, allowing the students to at least learn transducer placement for image acquisition, which is a good start to achieving competence.

All of our medical students also agreed that these modules are a good start to learning sonographic anatomy. Although majority of the students stated that the modules were adequate to learning sonographic technique and they had confidence in acquiring ultrasound images, their hands-on scores showed much room for improvement. The modules would be best utilized as an adjunct to hands-on learning and practice.

Nicholls et al. discusses the importance of psychomotor skills in ultrasound education and how they are the foundation of performing ultrasound examinations [13]. Therefore, these modules alone cannot allow medical students achieve mastery of POCUS. Furthermore, hands-on scanning is required to identify anatomical variations, tactile feedback, and pathology. Training should also progress beyond simulators to ultrasound machines used for patient care, so students develop familiarity and confidence in using these machines during their rotations. Previous investigations have identified that repetitive and active educational experiences, feedback, and embedding the education into the curriculum can maximize the learning experience [21].

Technical competency in ultrasound is built upon sustained deliberate practice, and our study shows that online education modules can provide this to a certain extent [22–24]. In this ever-evolving educational environment, this asynchronous learning resource can be a useful in the ultrasound education toolbox. Our results suggest that novices could potentially use this as an introductory learning tool and improve their skills with additional hands-on practice, which can be in the form of patients or simulators.

This project also shows promise in using asynchronous online learning modules for training medical personnel in military settings, where resources are scarce, and the environments may be austere.

With in-person ultrasound curricula transforming into virtual learning curricula, online learning modules with scanning technique can be integrated into tele-training. We believe our study has filled in a knowledge gap in the current literature. As discussed above, traditional methods of teaching ultrasound include both didactics (either in-person or asynchronous) and a hands-on training session. Because of the challenges we face during the COVID-19 pandemic, in-person trainings may not be feasible. If ultrasound novices can learn hands-on concepts by completing online modules, perhaps this can ultimately translate into a training model where medical providers can directly perform basic ultrasound examinations on patients after reviewing videos of online ultrasound educational content. We acknowledge this may only work for basic ultrasound applications and not for applications which require complex probe maneuvering techniques. We also recognize that this is only one learning platform out of innumerous resources. Programs may not be able to use SonoSim® because of cost, limitations with having a physical learning computer system, etc., and with virtual reality (VR) training tools expanding into the realm of medical education, virtual reality resources may become an educational tool staple.

### Limitations

This study is limited by its small sample size at a single institution, and the sample size was not determined a priori. Although this study was conducted early in the medical students’ first year of medical school, they have basic knowledge of anatomy and also an eagerness and interest in learning. These factors could have influenced the results, and we did not have a control group to control for these variables. Furthermore, not all students completed all of the modular components which could have underestimated the ability of SonoSim® modules to train. The hands-on assessment tool was not piloted nor validated prior to implementation. Although we established positive correlation between SonoSim® modules and hands-on acquisition of skills, further research exploring integration of SonoSim® modules with real-life hands-on training is needed. We did not investigate skills retention or effectiveness in the real-world clinical environment. Lastly, this study was not designed to assess overall impact or effectiveness of this simulation-based ultrasound system.

Realism and generalizability of these of these teaching techniques may be limited. Our study only evaluates one particular product and may not be generalizable.

## Conclusion

Our study findings suggest that ultrasound-naïve medical students can develop basic hands-on skills in image acquisition after reviewing online modules that teach ultrasound technique. This is particularly relevant during the COVID-19 pandemic, when teaching resources are greatly limited. Asynchronous learning has become the backbone of medical education curricula. Students are expected to have reviewed educational materials before short in-person trainings. Our study showed that there is value in online modules to teach procedures that require psychomotor training, such as performing ultrasound examinations.

## Supplementary Information

**Additional file 1.** Hands-on evaluation form used by the point-of-care ultrasound experts to evaluate medical students’ hands-on ultrasound performance.

**Additional file 2.** Medical student survey after hands-on portion completion.

**Additional file 3.** Data collected from study with descriptive statistical analysis.

**Additional file 4 **Data analysis and *p*-values.

**Additional file 5.** Links to SonoSim modules and descriptions.

## Data Availability

The datasets supporting the conclusions of this article are included within the article and its additional files.
