# Supplementary material for: Can ultrasound novices develop image acquisition skills after reviewing online ultrasound modules?
Source: BMC Med Educ. 2021 Mar 20;21:175. doi: 10.1186/s12909-021-02612-z (PMC7980807; doi:10.1186/s12909-021-02612-z)
Supplement: Supplementary file 1 — Additional file 1. Hands-on evaluation form used by the point-of-care ultrasound experts to evaluate medical students’ hands-on ultrasound performance. [file 12909_2021_2612_MOESM1_ESM.docx]

**Student Name: ______________________________________**

**MEDICAL STUDENT SONOSIM HANDS-ON EVALUATION FORM**

**Aorta/IVC**

Please ask the student to: Points:

Choose the most commonly used probe to ultrasound 0 1 2
the aorta and IVC (phased array - 1 point and curvilinear - 2 points)

Identify the vertebral body 0 1

Identify bowel gas 0 1

Show you abdominal aorta in short axis 0 1

Show you abdominal aorta in long axis 0 1 2

(1 point for oblique)

Show you the iliac bifurcation 0 1 2

(1 point if cannot see bifurcation, but in the correct region)

Show you the IVC in long axis 0 1 2

(1 point for oblique)

Show you the IVC in short axis 0 1

**Cardiac**

Please ask the student to:

Choose the most commonly used probe to ultrasound 0 1 2
the heart (curvilinear- 1pt and phased array- 2pts)

Show you a parasternal long axis view 0 1 2
(1 point if it is a partial PSL axis view)

Identify RV, septum, LV, and aorta/aortic outflow tract 0 1 2
(May give 1 point if able to identify at least 2 items)

Show you a subxiphoid view 0 1 2

(Give one point for a partial subxiphoid view)

Identify all 4 chambers on subxiphoid view 0 1 2
(1 point if they can identify at least 2 chambers)

Identify the liver 0 1

**Renal**

Please ask the student to:

Choose the most commonly used probe to ultrasound 0 1 2
the kidneys/bladder (phased array- 1pt and curvilinear- 2pts)

Identify the right kidney in long axis 0 1 2

(1 point if shown in short axis)

Identify the liver when scanning right kidney 0 1

Identify pyramids, medulla, and renal pelvis 0 1 2

(1 point if can identify at least one)

Show both transverse and sagittal views of the bladder 0 1 2
(1 point if can only show one view)

Identify the left kidney in short axis 0 1 2

(1 point if shown in long axis)

Identify the spleen when scanning the left kidney 0 1

Show blood flow in the renal pelvis 0 1

(turn on Color Doppler and place over pelvis)

**Superficial**

Please ask the student to:

Choose the most commonly used probe to ultrasound 0 1
superficial structures

Identify subcutaneous layer, muscle, fascial layer 0 1 2

(1 point if can identify at least one)

Identify tendon and bone 0 1 2

(1 point if can identify one)

Identify carotid artery and internal jugular vein 0 1 2

(1 point if can identify one)
