# Supplementary material for: Can ultrasound novices develop image acquisition skills after reviewing online ultrasound modules?
Source: BMC Med Educ. 2021 Mar 20;21:175. doi: 10.1186/s12909-021-02612-z (PMC7980807; doi:10.1186/s12909-021-02612-z)
Supplement: Supplementary file 2 — Additional file 2. Medical student survey after hands-on portion completion. [file 12909_2021_2612_MOESM2_ESM.docx]

**MEDICAL STUDENT SONOSIM ULTRASOUND STUDY SURVEY**

1.  To what extent do you agree or disagree with this statement: “SonoSim education modules are a good start to learning sonographic anatomy.” (Circle one.)

a. Strongly agree

b. Somewhat agree

c. Neither agree nor disagree

d. Somewhat disagree

e. Strongly disagree

2. To what extent do you agree or disagree with this statement: “SonoSim education modules are adequate to learning ultrasound scanning technique.” (Circle one.)

a. Strongly agree

b. Somewhat agree

c. Neither agree nor disagree

d. Somewhat disagree

e. Strongly disagree

3. Please rate your confidence level in acquiring ultrasound images. (Circle one number.)

Low                                                                                         High

1          2          3          4          5          6          7          8            9          10
