# Supplementary material for: Can ultrasound novices develop image acquisition skills after reviewing online ultrasound modules?
Source: BMC Med Educ. 2021 Mar 20;21:175. doi: 10.1186/s12909-021-02612-z (PMC7980807; doi:10.1186/s12909-021-02612-z)
Supplement: Supplementary file 4 — Additional file 4 Data analysis and p-values. [file 12909_2021_2612_MOESM4_ESM.docx]

. corr TotalScoreoutof100 TotalScoreHandson

(obs=20)

             | Tota~100 TotalS~n

-------------+------------------

TotalSco~100 |   1.0000

TotalScore~n |   0.6440   1.0000

. regress TotalScoreHandson TotalScoreoutof100

      Source |       SS       df       MS              Number of obs =      20

-------------+------------------------------           F(  1,    18) =   12.76

       Model |  973.026455     1  973.026455           Prob > F      =  0.0022

    Residual |  1373.10659    18  76.2836994           R-squared     =  0.4147

-------------+------------------------------           Adj R-squared =  0.3822

       Total |  2346.13304    19  123.480687           Root MSE      =  8.7341

------------------------------------------------------------------------------------

 TotalScoreHandson |      Coef.   Std. Err.      t    P>|t|     [95% Conf. Interval]

-------------------+----------------------------------------------------------------

TotalScoreoutof100 |    1.44028   .4032743     3.57   0.002     .5930323    2.287528

             _cons |  -44.41148   35.99484    -1.23   0.233    -120.0338    31.21088

------------------------------------------------------------------------------------

. corr AortaIVCScore AortaIVC

(obs=22)

             | AortaI~e AortaIVC

-------------+------------------

AortaIVCSc~e |   1.0000

    AortaIVC |   0.2564   1.0000

. regress AortaIVCScore AortaIVC

      Source |       SS       df       MS              Number of obs =      22

-------------+------------------------------           F(  1,    20) =    1.41

       Model |  221.778828     1  221.778828           Prob > F      =  0.2495

    Residual |  3152.58986    20  157.629493           R-squared     =  0.0657

-------------+------------------------------           Adj R-squared =  0.0190

       Total |  3374.36869    21  160.684223           Root MSE      =  12.555

------------------------------------------------------------------------------

AortaIVCSc~e |      Coef.   Std. Err.      t    P>|t|     [95% Conf. Interval]

-------------+----------------------------------------------------------------

    AortaIVC |   .4899549   .4130616     1.19   0.249    -.3716765    1.351586

       _cons |    40.2624   37.36542     1.08   0.294     -37.6805    118.2053

------------------------------------------------------------------------------

. corr CardiacScore Cardiac

(obs=21)

             | Cardia~e  Cardiac

-------------+------------------

CardiacScore |   1.0000

     Cardiac |   0.1356   1.0000

. regress CardiacScore Cardiac

      Source |       SS       df       MS              Number of obs =      21

-------------+------------------------------           F(  1,    19) =    0.36

       Model |  113.851584     1  113.851584           Prob > F      =  0.5579

    Residual |  6080.56006    19  320.029477           R-squared     =  0.0184

-------------+------------------------------           Adj R-squared = -0.0333

       Total |  6194.41165    20  309.720582           Root MSE      =  17.889

------------------------------------------------------------------------------

CardiacScore |      Coef.   Std. Err.      t    P>|t|     [95% Conf. Interval]

-------------+----------------------------------------------------------------

     Cardiac |   .3267045   .5477478     0.60   0.558    -.8197447    1.473154

       _cons |   52.52638   48.54162     1.08   0.293    -49.07239    154.1251

------------------------------------------------------------------------------

. corr RenalScore Renal

(obs=22)

             | RenalS~e    Renal

-------------+------------------

  RenalScore |   1.0000

       Renal |   0.1916   1.0000

. regress RenalScore Renal

      Source |       SS       df       MS              Number of obs =      22

-------------+------------------------------           F(  1,    20) =    0.76

       Model |  219.493102     1  219.493102           Prob > F      =  0.3931

    Residual |   5762.2175    20  288.110875           R-squared     =  0.0367

-------------+------------------------------           Adj R-squared = -0.0115

       Total |   5981.7106    21  284.843362           Root MSE      =  16.974

------------------------------------------------------------------------------

  RenalScore |      Coef.   Std. Err.      t    P>|t|     [95% Conf. Interval]

-------------+----------------------------------------------------------------

       Renal |   .5506581   .6308868     0.87   0.393    -.7653487    1.866665

       _cons |   34.88709    53.8904     0.65   0.525    -77.52632    147.3005

------------------------------------------------------------------------------

. corr SuperficialScore SoftTissueSuperficial

(obs=23)

             | Superf~e SoftTi~l

-------------+------------------

Superficia~e |   1.0000

SoftTissue~l |   0.2285   1.0000

. regress SuperficialScore SoftTissueSuperficial

      Source |       SS       df       MS              Number of obs =      23

-------------+------------------------------           F(  1,    21) =    1.16

       Model |  125.106186     1  125.106186           Prob > F      =  0.2943

    Residual |  2270.63472    21  108.125463           R-squared     =  0.0522

-------------+------------------------------           Adj R-squared =  0.0071

       Total |  2395.74091    22  108.897314           Root MSE      =  10.398

---------------------------------------------------------------------------------------

     SuperficialScore |      Coef.   Std. Err.      t    P>|t|     [95% Conf. Interval]

----------------------+----------------------------------------------------------------

SoftTissueSuperficial |   .3197158   .2972273     1.08   0.294    -.2984022    .9378338

                _cons |   64.32378   26.90259     2.39   0.026     8.376782    120.2708

---------------------------------------------------------------------------------------

.

.

. corr CardiacScore CardiacDifficulty15

(obs=25)

             | Cardia~e Cardi~15

-------------+------------------

CardiacScore |   1.0000

CardiacDi~15 |  -0.4431   1.0000

. regress CardiacScore CardiacDifficulty15

      Source |       SS       df       MS              Number of obs =      25

-------------+------------------------------           F(  1,    23) =    5.62

       Model |  1905.68789     1  1905.68789           Prob > F      =  0.0265

    Residual |  7800.09723    23  339.134662           R-squared     =  0.1963

-------------+------------------------------           Adj R-squared =  0.1614

       Total |  9705.78512    24  404.407713           Root MSE      =  18.416

-------------------------------------------------------------------------------------

       CardiacScore |      Coef.   Std. Err.      t    P>|t|     [95% Conf. Interval]

--------------------+----------------------------------------------------------------

CardiacDifficulty15 |  -7.486631   3.158251    -2.37   0.027    -14.01997   -.9532912

              _cons |    101.369   10.16545     9.97   0.000     80.34015    122.3978

-------------------------------------------------------------------------------------

. corr RenalScore RenalDifficulty15

(obs=25)

             | RenalS~e Renal~15

-------------+------------------

  RenalScore |   1.0000

RenalDiff~15 |  -0.5560   1.0000

. regress RenalScore RenalDifficulty15

      Source |       SS       df       MS              Number of obs =      25

-------------+------------------------------           F(  1,    23) =   10.29

       Model |  2344.59085     1  2344.59085           Prob > F      =  0.0039

    Residual |   5238.8411    23    227.7757           R-squared     =  0.3092

-------------+------------------------------           Adj R-squared =  0.2791

       Total |  7583.43195    24  315.976331           Root MSE      =  15.092

-----------------------------------------------------------------------------------

       RenalScore |      Coef.   Std. Err.      t    P>|t|     [95% Conf. Interval]

------------------+----------------------------------------------------------------

RenalDifficulty15 |  -9.060547   2.824064    -3.21   0.004    -14.90257   -3.218525

            _cons |   106.2379   8.358467    12.71   0.000     88.94707    123.5287

-----------------------------------------------------------------------------------

. corr SuperficialScore SuperficialDifficulty15

(obs=24)

             | Superf~e Super~15

-------------+------------------

Superficia~e |   1.0000

Superfici~15 |  -0.6175   1.0000

. regress SuperficialScore SuperficialDifficulty15

      Source |       SS       df       MS              Number of obs =      24

-------------+------------------------------           F(  1,    22) =   13.56

       Model |  1971.61623     1  1971.61623           Prob > F      =  0.0013

    Residual |  3198.45179    22  145.384172           R-squared     =  0.3814

-------------+------------------------------           Adj R-squared =  0.3532

       Total |  5170.06803    23  224.785566           Root MSE      =  12.058

-----------------------------------------------------------------------------------------

       SuperficialScore |      Coef.   Std. Err.      t    P>|t|     [95% Conf. Interval]

------------------------+----------------------------------------------------------------

SuperficialDifficulty15 |  -10.09852   2.742238    -3.68   0.001    -15.78558   -4.411468

                  _cons |   108.9901   5.597571    19.47   0.000      97.3815    120.5988

-----------------------------------------------------------------------------------------

. corr AortaScore AortadifficultyEasiest15Mo

(obs=25)

             | AortaS~e Aortad~o

-------------+------------------

  AortaScore |   1.0000

Aortadiffi~o |  -0.4455   1.0000

. regress AortaScore AortadifficultyEasiest15Mo

      Source |       SS       df       MS              Number of obs =      25

-------------+------------------------------           F(  1,    23) =    5.70

       Model |  .127021701     1  .127021701           Prob > F      =  0.0256

    Residual |  .512978299    23  .022303404           R-squared     =  0.1985

-------------+------------------------------           Adj R-squared =  0.1636

       Total |         .64    24  .026666667           Root MSE      =  .14934

--------------------------------------------------------------------------------------------

  AortaScore |      Coef.   Std. Err.      t    P>|t|     [95% Conf. Interval]

---------------------------+----------------------------------------------------------------

AortadifficultyEasiest15Mo |  -.0702208   .0294247    -2.39   0.026    -.1310906   -.0093511

                     _cons |   .9720497   .0832257    11.68   0.000     .7998841    1.144215

--------------------------------------------------------------------------------------------

. corr IVCScore IVCDifficulty15

(obs=25)

             | IVCScore IVCDi~15

-------------+------------------

    IVCScore |   1.0000

IVCDiffic~15 |  -0.5660   1.0000

. regress IVCScore IVCDifficulty15

      Source |       SS       df       MS              Number of obs =      25

-------------+------------------------------           F(  1,    23) =   10.84

       Model |  .435630252     1  .435630252           Prob > F      =  0.0032

    Residual |  .924369748    23  .040189989           R-squared     =  0.3203

-------------+------------------------------           Adj R-squared =  0.2908

       Total |        1.36    24  .056666667           Root MSE      =  .20047

---------------------------------------------------------------------------------

       IVCScore |      Coef.   Std. Err.      t    P>|t|     [95% Conf. Interval]

----------------+----------------------------------------------------------------

IVCDifficulty15 |  -.1512605   .0459436    -3.29   0.003    -.2463022   -.0562188

          _cons |   1.184874   .1121627    10.56   0.000     .9528476      1.4169

---------------------------------------------------------------------------------

.

. corr goodstart TotalScoreoutof100

(obs=20)

             | goodst~t Tota~100

-------------+------------------

   goodstart |   1.0000

TotalSco~100 |  -0.4915   1.0000

. regress goodstart TotalScoreoutof100

      Source |       SS       df       MS              Number of obs =      20

-------------+------------------------------           F(  1,    18) =    5.73

       Model |  .229480346     1  .229480346           Prob > F      =  0.0277

    Residual |  .720519654    18   .04002887           R-squared     =  0.2416

-------------+------------------------------           Adj R-squared =  0.1994

       Total |         .95    19         .05           Root MSE      =  .20007

------------------------------------------------------------------------------------

         goodstart |      Coef.   Std. Err.      t    P>|t|     [95% Conf. Interval]

-------------------+----------------------------------------------------------------

TotalScoreoutof100 |  -.0221186   .0092379    -2.39   0.028    -.0415266   -.0027106

             _cons |   3.021319   .8245387     3.66   0.002     1.289028    4.753611

------------------------------------------------------------------------------------

. corr goodstart TotalScoreHandson

(obs=25)

             | goodst~t TotalS~n

-------------+------------------

   goodstart |   1.0000

TotalScore~n |  -0.2571   1.0000

. regress goodstart TotalScoreHandson

      Source |       SS       df       MS              Number of obs =      25

-------------+------------------------------           F(  1,    23) =    1.63

       Model |  .174473628     1  .174473628           Prob > F      =  0.2148

    Residual |  2.46552637    23  .107196799           R-squared     =  0.0661

-------------+------------------------------           Adj R-squared =  0.0255

       Total |        2.64    24         .11           Root MSE      =  .32741

-----------------------------------------------------------------------------------

        goodstart |      Coef.   Std. Err.      t    P>|t|     [95% Conf. Interval]

------------------+----------------------------------------------------------------

TotalScoreHandson |  -.0065352   .0051225    -1.28   0.215    -.0171319    .0040615

            _cons |   1.654363   .4239418     3.90   0.001     .7773731    2.531354

-----------------------------------------------------------------------------------

.

. corr adequate TotalScoreoutof100

(obs=20)

             | adequate Tota~100

-------------+------------------

    adequate |   1.0000

TotalSco~100 |  -0.0117   1.0000

. regress adequate TotalScoreoutof100

      Source |       SS       df       MS              Number of obs =      20

-------------+------------------------------           F(  1,    18) =    0.00

       Model |  .001199201     1  .001199201           Prob > F      =  0.9610

    Residual |   8.7988008    18  .488822267           R-squared     =  0.0001

-------------+------------------------------           Adj R-squared = -0.0554

       Total |         8.8    19  .463157895           Root MSE      =  .69916

------------------------------------------------------------------------------------

          adequate |      Coef.   Std. Err.      t    P>|t|     [95% Conf. Interval]

-------------------+----------------------------------------------------------------

TotalScoreoutof100 |  -.0015989    .032282    -0.05   0.961    -.0694209     .066223

             _cons |   1.742505   2.881376     0.60   0.553    -4.311041    7.796051

------------------------------------------------------------------------------------

. corr adequate TotalScoreHandson

(obs=25)

             | adequate TotalS~n

-------------+------------------

    adequate |   1.0000

TotalScore~n |  -0.0270   1.0000

. regress adequate TotalScoreHandson

      Source |       SS       df       MS              Number of obs =      25

-------------+------------------------------           F(  1,    23) =    0.02

       Model |  .010980724     1  .010980724           Prob > F      =  0.8980

    Residual |  15.0290193    23  .653435621           R-squared     =  0.0007

-------------+------------------------------           Adj R-squared = -0.0427

       Total |       15.04    24  .626666667           Root MSE      =  .80835

-----------------------------------------------------------------------------------

         adequate |      Coef.   Std. Err.      t    P>|t|     [95% Conf. Interval]

------------------+----------------------------------------------------------------

TotalScoreHandson |  -.0016395   .0126472    -0.13   0.898    -.0278021    .0245232

            _cons |   1.854056   1.046687     1.77   0.090    -.3111801    4.019293

-----------------------------------------------------------------------------------

.

. corr confident TotalScoreoutof100

(obs=20)

             | confid~t Tota~100

-------------+------------------

   confident |   1.0000

TotalSco~100 |   0.2845   1.0000

. regress confident TotalScoreoutof100

      Source |       SS       df       MS              Number of obs =      20

-------------+------------------------------           F(  1,    18) =    1.59

       Model |  2.42838108     1  2.42838108           Prob > F      =  0.2241

    Residual |  27.5716189    18  1.53175661           R-squared     =  0.0809

-------------+------------------------------           Adj R-squared =  0.0299

       Total |          30    19  1.57894737           Root MSE      =  1.2376

------------------------------------------------------------------------------------

         confident |      Coef.   Std. Err.      t    P>|t|     [95% Conf. Interval]

-------------------+----------------------------------------------------------------

TotalScoreoutof100 |    .071952   .0571452     1.26   0.224    -.0481055    .1920096

             _cons |   .5872751   5.100577     0.12   0.910    -10.12864    11.30319

------------------------------------------------------------------------------------

. corr confident TotalScoreHandson

(obs=25)

             | confid~t TotalS~n

-------------+------------------

   confident |   1.0000

TotalScore~n |   0.3162   1.0000

. regress confident TotalScoreHandson

      Source |       SS       df       MS              Number of obs =      25

-------------+------------------------------           F(  1,    23) =    2.55

       Model |  3.85469392     1  3.85469392           Prob > F      =  0.1236

    Residual |  34.7053061    23  1.50892635           R-squared     =  0.1000

-------------+------------------------------           Adj R-squared =  0.0608

       Total |       38.56    24  1.60666667           Root MSE      =  1.2284

-----------------------------------------------------------------------------------

        confident |      Coef.   Std. Err.      t    P>|t|     [95% Conf. Interval]

------------------+----------------------------------------------------------------

TotalScoreHandson |   .0307175   .0192188     1.60   0.124    -.0090395    .0704746

            _cons |   4.248305   1.590557     2.67   0.014     .9579868    7.538624

-----------------------------------------------------------------------------------
