# Supplementary material for: Can ultrasound novices develop image acquisition skills after reviewing online ultrasound modules?
Source: BMC Med Educ. 2021 Mar 20;21:175. doi: 10.1186/s12909-021-02612-z (PMC7980807; doi:10.1186/s12909-021-02612-z)
Supplement: Supplementary file 5 — Additional file 5. Links to SonoSim modules and descriptions. [file 12909_2021_2612_MOESM5_ESM.docx]

Links to more detailed descriptions of each of the SonoSim modules reviewed by students:

Aorta/IVC:

<https://sonosim.com/shop/aortaivc-core-clinical/>

Cardiology:
<https://sonosim.com/shop/cardiology-core-clinical/>

Renal:

<https://sonosim.com/shop/renal-core-clinical/>

Soft Tissue (Superficial):

<https://sonosim.com/shop/soft-tissue-core-clinical/>
